# Prevalence and outcomes of end-stage kidney disease in patients with systemic lupus erythematous: a population-based study

**DOI:** 10.1007/s00296-023-05409-z

**Published:** 2023-08-14

**Authors:** Chunhuan Lao, Philippa Van Dantzig, Douglas White, Kannaiyan Rabindranath, Donna Foxall, Ross Lawrenson

**Affiliations:** 1 Medical Research Centre, The University of Waikato, Private Bag 3105, Hamilton, 3240 New Zealand; 2 Rheumatology Department, Waikato Hospital, Hamilton, New Zealand; 3 Renal Unit, Waikato Hospital, Hamilton, New Zealand; 4 Te Huataki Waiora, School of Health, The University of Waikato, Hamilton, New Zealand; 5 Strategy and Funding, Waikato Hospital, Hamilton, New Zealand

**Keywords:** Systemic lupus erythematosus, End-stage kidney disease, ANZDATA, Period prevalence, Mortality

## Abstract

This study aims to examine the prevalence and outcomes of end-stage kidney disease (ESKD) among systemic lupus erythematosus (SLE) patients. SLE patients identified from the national administrative datasets were linked to the Australia and New Zealand Dialysis and Transplant Registry (ANZDATA) to identify the ESKD cases. Period prevalence of ESKD among SLE patients was calculated. The risk of developing ESKD by ethnicity was explored with Cox Proportional Hazards model. The adjusted hazard ratio (HR) of all-cause mortality for Māori, Pacific, Asian compared to European/others was estimated. Of the 2837 SLE patients, 210 (7.4%) developed ESKD. The average period prevalence of ESKD among SLE patients was 5.7%. Men had twice the prevalence rate of ESKD than women (10.0% vs 5.2%). Māori and Pacific had higher prevalence rate than Asian and European/others (9.4%, 9.8% vs 4.4% and 3.8%). The adjusted HR of developing ESKD for men compared to women was 3.37 (95% CI 1.62–7.02). The adjusted HR of developing ESKD for Māori and Pacific compared to European/others was 4.63 (95% CI 1.61–13.29) and 4.66 (95% CI 1.67–13.00), respectively. Compared to European/others, Māori had an HR of 2.17 (95% CI 1.18–4.00) for all-cause mortality. SLE patients had a high prevalence rate of ESKD. Men, Māori, and Pacific patients with SLE were more likely to develop ESKD. Māori patients with ESKD had poorer survival than other patients. Interventions are needed to reduce the risk of ESKD and to improve the survival of ESKD patients for the disadvantaged groups.

## Introduction

Systemic lupus erythematosus (SLE) is the prototypic autoimmune disease. The immune system attacks its own tissue and organs, including joints, skin, central nervous system, lungs, kidneys, gastro-intestinal tract, cardiovascular system, and bone marrow [1]. Involvement of the kidneys in SLE is termed lupus nephritis (LN) and can present with hematuria, proteinuria, hypertension, renal impairment, or rapidly progressive crescentic glomerulonephritis with kidney failure. There are 6 classes of lupus nephritis and classically, classes III and IV are most concerning and require treatment with immunosuppression [2]. Approximately, 30–60% of patients with SLE will progress to LN and 10–30% of those with class III LN and above will develop end-stage kidney disease (ESKD) within 15 years after LN diagnosis [3, 4].

ESKD is defined as a glomerular filtration rate (GFR) of less than 15 mL/min [5]. ESKD can cause a wide range of signs and symptoms, including wastes, fluids, electrolytes, and minerals to accumulate in the body. Patients with ESKD cannot survive without dialysis or renal transplant. ESKD is a significant cause of reduced quality of life and premature mortality [5]. SLE-related ESKD is associated with poorer outcomes than non-SLE ESKD. Data from the Australia and New Zealand Dialysis and Transplant Registry (ANZDATA) reported that patients with SLE-related ESKD had an adjusted survival rate of 40% at 10 years compared to 52% for non-SLE ESKD [6].

The incidence of SLE and the risk of progression to LN differs by ethnicity. In New Zealand, Pacific, Māori and Asian people had higher prevalence of SLE than European/others (176.2, 83.7, 72.2 versus 48.5 per 100,000) [7]. A New Zealand study in 2007 demonstrated that 56 (32.9%) out of 170 SLE patients developed renal disease, but the severity of the renal disease (e.g., ESKD) was not reported [8]. This paper reported that Māori and Pacific patients with SLE had a higher risk of developing LN, with an odds ratio of 8.47 and 3.11 compared to others [8]. The development of ESKD is an important outcome measurement of SLE management, and this has not been researched in New Zealand. This study aims to examine the prevalence and outcomes of ESKD among SLE patients, and to identify the disparities between ethnic groups in New Zealand. This is the first national study in New Zealand providing data on the burden of ESKD caused by SLE.

## Methods

SLE patients were identified using the ICD-10 code “M32” from the National Minimum Dataset (NMDS) and the Mortality Collection (coded death records) and using the key words “systemic lupus erythematosus” from the Death Certificates (uncoded death records). The first date in the NMDS for an inpatient event with an ICD-10 code of “M32” or the first date from the National Non-admitted Patient Collection (NNAPC) for an outpatient event in the Rheumatology department or Renal Service, was considered as the date of first identification of SLE. These SLE patients were then linked to the ANZDATA through the patients’ National Health Index (NHI) numbers, a unique identifier for people using health and disability Services in New Zealand. The study period was between 1 January 2005 and 31 December 2021. The NMDS records inpatient and day patient events, and the NNAPC stores outpatient events and emergency department events. These two datasets cover all public hospitals and over 90% of private hospitals. The NMDS and the NNAPC start collecting data from 2005, and the NNAPC does not include ICD codes. The Mortality Collection contains date and cause of deaths coded in ICD-10 code, and the Death Certificates collects more up-to-date death records not included in the Mortality Collection yet. The ANZDATA is a clinical quality registry that collects the treatments and outcomes of end-stage kidney failure in New Zealand and Australia since 1977. It includes but not excludes the primary disease causing ESKD and the first date of ESKD treatment. The date of ESKD diagnosis was not recorded, and the first date of ESKD treatment was used as the date of ESKD diagnosis.

We validated the date of SLE diagnosis by comparing the data from national administrative datasets with the medical records in the Waikato Hospital for patients living in this region. For patients identified in 2010–2021, the dates of SLE identification were more accurate than patients identified in earlier years, with 78.4% of patients identified in 2010–2021 having an accurate date of first SLE identification and 11.4% of patients having a gap of 1–5 years between the two dates. Therefore, the data analysis using the date of SLE diagnosis only included patients identified in 2010–2021.

The primary disease of ESKD was categorized into (1) SLE, (2) diabetes and (3) others. The proportion of ESKD by primary disease was estimated, and compared by ethnicity (Māori, Pacific, Asian, and European/others). Other patient characteristics including age at ESKD diagnosis, gender (women and men), socioeconomic status and comorbidities were also compared by ethnic group. Socioeconomic deprivation was defined using the New Zealand Index of Deprivation 2018 (NZDep 2018) analyzed as quintile, from 1 (least deprived) to 5 (most deprived) [9]. Comorbidities were identified from the NMDS and were examined with Charlson Comorbidity Index (CCI) score which was divided into four groups: 0, 1, 2, and 3 + [10]. Cumulative incidence of ESKD over the whole follow-up period was estimated. Period prevalence of ESKD over 12 months among SLE patients in 2010–2021 was calculated and was compared by gender, ethnicity, socioeconomic status, and age in that specific year (< 20, 20–39, 40–59, 60–79, and 80 + years). Period prevalence is the proportion of a population that has the characteristic or a particular disease at any point during a given time period of interest. In this study, the time period was one year from 1st January to 31st December in that particular year.

The risk of developing ESKD for Māori, Pacific, Asian compared to European/others was explored with Cox Proportional Hazards model using the SLE patients identified in 2010–2021. The hazard ratio (HR) and the 95% confidence interval (CI) were estimated, after adjustment for gender, socioeconomic status and age at SLE diagnosis. Among ESKD patients, HR of all-cause mortality for Māori, Pacific, Asian compared to European/others was explored was also estimated, after adjustment for gender, socioeconomic status, age at ESKD diagnosis, and comorbidities. The censor date for the risk of developing ESKD was 31 December 2021 (last date in the ANZDATA) and the censor date for all-cause mortality was 31 December 2022 (last date in the Mortality Collection and Death Certificates).

All data analyses were performed in IBM SPSS 29 (New York, United States). Ethics approval for the study was granted through the Northern B Health and Disability Ethics Committee (reference: 2022 EXP 13741). The data reported here have been supplied by the Australia and New Zealand Dialysis and Transplant Registry. The interpretation and reporting of these data are the responsibility of the authors and in no way should be seen as an official policy or interpretation of the Australia and New Zealand Dialysis and Transplant Registry.

## Results

During the study period, 2837 patients with SLE were identified and 210 (7.4%, cumulative incidence) of them developed ESKD up till the end of 2021, including 168 (80%) women and 42 (20%) men (Table 1). Most of the ESKD cases were caused by SLE (182, 86.7%) and 13 (6.2%) were caused by diabetes. The mean age at ESKD was 40 years, and 54.3% of patients had ESKD under the age of 40 years old. Māori and Pacific patients with ESKD were more likely to be younger, with 64.8% and 60.3% of them being less than 40 years compared to 40.7% for Asian and 46.5% for European/others (*p*-value < 0.05). They were also more likely to live in the most deprived areas, with 55.6% of Māori and 51.8% of Pacific patients with ESKD living in the deprivation quintile 5 areas compared to 14.8% of Asian and 8.5% of European/others (*p*-value < 0.05). Pacific patients were half as likely to be men than other ethnic groups (12.1% versus 21.1% to 25.9%; *p*-value < 0.05).

**Table 1:** Characteristics of patients with ESKD

Characteristics	Asian		European /others		Māori		Pacific		Total	
Primary ESKD disease										
SLE	21	77.8%	62	87.3%	47	87.0%	48	82.8%	182	86.7%
Diabetes	2	7.4%	2	2.8%	4	7.4%	5	8.6%	13	6.2%
Others	3	11.1%	6	8.5%	3	5.6%	3	5.2%	15	7.1%
Age at ESKD diagnosis (years)										
0–19	0		2	2.8%	10	18.5%	6	10.3%	18	8.6%
20–29	4	14.8%	13	18.3%	12	22.2%	13	22.4%	42	20.0%
30–39	7	25.9%	18	25.4%	13	24.1%	16	27.6%	54	25.7%
40–49	6	22.2%	9	12.7%	9	16.7%	8	13.8%	32	15.2%
50–59	9	33.3%	15	21.1%	5	9.3%	9	15.5%	38	18.1%
60–69	1	3.7%	11	15.5%	4	7.4%	3	5.2%	19	9.0%
70–79	0		3	4.2%	1	1.9%	3	5.2%	7	3.3%
Gender										
Women	21	77.8%	56	78.9%	40	74.1%	51	87.9%	168	80.0%
Men	6	22.2%	15	21.1%	14	25.9%	7	12.1%	42	20.0%
Deprivation quintile										
1 (least deprived)	9	33.3%	17	23.9%	1	1.9%	1	1.7%	28	13.3%
2	3	11.1%	14	19.7%	7	13.0%	7	12.1%	31	14.8%
3	6	22.2%	18	25.4%	8	14.8%	4	6.9%	36	17.1%
4	5	18.5%	16	22.5%	8	14.8%	15	25.9%	44	21.0%
5 (most deprived)	4	14.8%	6	8.5%	30	55.6%	30	51.7%	70	33.3%
Unknown							1		1	
Charlson comorbidity score										
0	20	74.1%	43	60.6%	33	61.1%	35	60.3%	131	62.4%
1	6	22.2%	23	32.4%	16	29.6%	14	24.1%	59	28.1%
2	0		1	1.4%	5	9.3%	6	10.3%	12	5.7%
3 +	1	3.7%	4	5.6%	0		3	5.2%	8	3.8%
Total	27	12.9%	71	33.8%	54	25.7%	58	27.6%	210	100.0%

The average period prevalence of ESKD among SLE patients in 2010–2021 was 5.7% (Table 2). Men had twice the prevalence rate of ESKD than women (10.0% vs 5.2%, *p*-value < 0.05), and Māori and Pacific patients had more than twice the prevalence rate of ESKD than Asian and European/others (9.4%, 9.8% vs 4.4% and 3.8%, *p*-value < 0.05). The prevalence of ESKD among SLE patients first increased with age from 2.0% for those aged less than 20 years old to 6.8% for patients aged 20–39 years, and then decreased with age to 6.5% for those aged 40–59 years, 4.3% for patients aged 60–79 years and 1.6% for those aged 80 years or older (*p*-value < 0.05). The prevalence of ESKD has slightly increased with time over the study period, from 5.5% in 2010 to 6.0%.

**Table 2:** Period prevalence of ESKD among SLE patients

Subgroup	2010	2011	2012	2013	2014	2015	2016	2017	2018	2019	2020	2021	Average
Gender													
Women	5.1%	5.3%	4.9%	4.9%	5.0%	5.0%	5.0%	5.3%	5.3%	5.2%	5.5%	5.4%	5.2%
Men	8.9%	8.8%	9.1%	9.2%	8.7%	9.6%	10.7%	10.8%	10.9%	11.1%	11.2%	10.5%	10.0%
Ethnicity													
Asian	2.6%	3.4%	3.2%	4.3%	4.2%	4.6%	4.6%	5.2%	4.5%	4.6%	5.4%	5.8%	4.4%
European/others	4.1%	4.2%	3.8%	3.6%	3.5%	3.4%	3.5%	3.6%	3.7%	3.8%	3.9%	4.0%	3.8%
Māori	9.3%	8.9%	10.3%	9.2%	9.6%	9.4%	10.0%	10.1%	10.3%	9.3%	8.9%	7.9%	9.4%
Pacific	9.5%	9.5%	7.9%	8.6%	9.6%	9.7%	9.9%	10.3%	10.5%	10.7%	11.1%	10.3%	9.8%
Deprivation quintile													
1 (least deprived)	4.8%	5.5%	4.8%	5.0%	4.7%	4.0%	4.2%	4.7%	4.9%	4.8%	5.7%	6.3%	4.9%
2	5.0%	5.4%	5.6%	6.1%	5.0%	5.9%	6.1%	5.9%	6.1%	5.8%	5.7%	5.7%	5.7%
3	6.2%	5.9%	5.4%	4.9%	5.6%	4.9%	5.0%	5.8%	5.6%	5.5%	5.4%	5.3%	5.5%
4	3.1%	2.8%	3.0%	3.1%	3.6%	4.5%	4.6%	4.7%	4.3%	4.9%	5.5%	5.1%	4.1%
5 (most deprived)	8.2%	8.6%	8.0%	7.6%	6.7%	7.7%	8.1%	8.3%	8.5%	8.2%	8.1%	7.6%	8.0%
Age (years) in that specific year													
< 20	6.3%	5.1%	5.1%	1.4%	1.4%	2.6%	0.0%	0.0%	0.0%	0.0%	1.3%	1.2%	2.0%
20–39	6.2%	6.1%	6.2%	6.5%	6.2%	6.5%	7.0%	7.0%	7.2%	7.3%	7.7%	7.3%	6.8%
40–59	5.8%	6.2%	6.0%	6.3%	6.4%	6.2%	5.9%	6.9%	7.0%	6.7%	7.0%	7.1%	6.5%
60–79	3.9%	4.5%	3.6%	3.4%	4.2%	4.1%	5.2%	4.9%	4.5%	4.8%	4.4%	4.3%	4.3%
80 +	2.3%	2.0%	2.1%	1.7%	1.6%	1.8%	1.5%	0.0%	0.0%	1.4%	2.7%	2.6%	1.6%
Overall	5.5%	5.7%	5.4%	5.4%	5.5%	5.5%	5.7%	6.0%	6.0%	5.9%	6.2%	6.0%	5.7%

Among the 1145 SLE patients identified in 2010–2021, 34 (3.0%) developed ESKD, with a median follow-up time of 6.5 years. The risk of developing ESKD was higher for men than women, with an HR of 3.37 (95% CI 1.62–7.02, *p*-value = 0.001) after adjustment for age, ethnicity, and socioeconomic status (Table 3). Māori and Pacific patients were more likely to have ESKD, with an adjusted HR of 4.63 (95% CI: 1.61–13.29, *p*-value = 0.004) and 4.66 (95% CI 1.67–13.00, *p*-value = 0.003) compared to European/others. We could not find any significant impact of age and socioeconomic status on risk of ESKD, but that may be due to the relatively short follow-up time for these patients.

**Table 3:** Adjusted hazard ratio of developing ESKD among SLE patients identified in 2010–21

Factor	Adjusted hazard ratio (95% CI)	*p*-value
Gender		
Women	Reference	
Men	**3.37 (1.62–7.02)**	**0.001**
Ethnicity		
European/others	Reference	
Asian	1.70 (0.52–5.57)	0.380
Māori	**4.63 (1.61–13.29)**	**0.004**
Pacific	**4.66 (1.67–13.00)**	**0.003**
Deprivation quintile		
1 (least deprived)	Reference	
2	0.40 (0.07–2.18)	0.289
3	0.80 (0.21–3.04)	0.746
4	1.12 (0.35–3.62)	0.852
5 (most deprived)	0.70 (0.21–2.37)	0.567
Age at SLE diagnosis (continuous)	0.99 (0.97–1.01)	0.304

Bold values indicate statistically significant

Of the 210 ESKD patients, 92 deceased during the follow-up period, with a mean age at death of 51.8 years (standard deviation: 15.6 years) (Table 4). The 5-year and 10-year all-cause survival of ESKD patients was 78.6% (95% CI 73.0–84.6%) and 62.5% (95% CI 55.6–70.2%), respectively. Māori patients and patients aged 60–79 years old had the lowest 10-year survival. The mean age at death was youngest for Māori (47.1 years), followed by Pacific (52.1 years) and European/others (54.0 years), and was highest for Asian (58.6 years). The mean age at death was only 21 years for patients diagnosed with ESKD before the age of 20 years, 42 years for patients diagnosed at the age of 20–39 years, 56.7 years for those diagnosed at 40–59 years and 70.3 years for those diagnosed at 60–79 years. Compared to European/others, Māori patients were more likely to die, with an HR of 2.17 (95% CI: 1.18–4.00, *p*-value = 0.013) for all-cause mortality, after adjustment for age, gender, socioeconomic status, and comorbidities (Table 5). The adjusted HR for all-cause mortality for age was 1.05 (95% CI: 1.03–1.07, *p*-value < 0.001).

**Table 4:** All-cause survival and age at death by subgroup

Subgroup	All-cause survival (95% CI)		Age at death (years)	
	5-year	10-year	Mean	Standard deviation
Gender				
Women	77.2% (70.9–84.1%)	60.3% (52.6–69.1%)	51.5	15.6
Men	84.2% (73.3–96.7%)	71.2% (57.4–88.3%)	53.4	17.6
Ethnicity				
Asian	84.6% (71.7–99.7%)	72.4% (55.1–95.1%)	58.6	9.7
European/Others	73.3% (63.4–84.7%)	66.1% (55.3–78.9%)	54.0	16.0
Māori	78.9% (68.6–90.8%)	54.7% (52.2–70.9%)	47.1	15.3
Pacific	82.3% (72.4–93.6%)	62.4% (49.5–78.8%)	52.1	16.5
Age at ESKD diagnosis				
< 20	76.6% (58.8–99.7%)	68.9% (49.3–96.4%)	21.0	3.3
20–39	92.5% (84.7–100.0%)	82.6% (70.7–96.5%)	42.1	9.6
40–59	81.5% (71.3–93.2%)	69.7% (57.5–84.5%)	56.7	8.3
60–79	87.3% (76.4–99.7%)	47.8% (32.3–70.6%)	70.3	6.7
Overall	78.6% (73.0–84.6%)	62.5% (55.6–70.2%)	51.8	15.6

**Table 5:** Adjusted hazard ratio of all-cause survival among ESKD patients

Factor	Adjusted hazard ratio (95% CI)	*p*-value
Gender		
Women	Reference	
Men	0.71 (0.40–1.26)	0.240
Ethnicity		
European/others	Reference	
Asian	1.20 (0.55–2.62)	0.643
Māori	**2.17 (1.18–4.00)**	**0.013**
Pacific	1.36 (0.70–2.64)	0.360
Deprivation quintile		
1 (least deprived)	Reference	
2	0.78 (0.30–2.02)	0.612
3	1.33 (0.56–3.12)	0.517
4	1.78 (0.77–4.11)	0.176
5 (most deprived)	1.08 (0.45–2.60)	0.860
Age at ESKD diagnosis (continuous)	**1.05 (1.03–1.07)**	** < 0.001**
Charlson comorbidity score		
0	Reference	
1	0.96 (0.60–1.55)	0.866
2	0.76 (0.32–1.84)	0.547
3 +	2.26 (0.92–5.56)	0.075

Bold values indicate statistically significant

## Discussion

This study showed that the average prevalence of ESKD among SLE patients was 5.7%, which was 58 times the prevalence in the general population in New Zealand in 2018 (985 per 1,000,000) reported by the ANZDATA registry [11]. A Korean study also demonstrated a crude HR of 18.6 for ESKD among SLE patients compared to the general population [12]. The cumulative incidence of ESKD among newly diagnosed SLE patients was 3.0% (34/1145) in New Zealand, with a median follow-up time of 6.5 years. This rate was comparable to the cumulative incidence rates reported in Taiwan and Japan which was 2.5% over 6–8 years of follow-up [13] and 3.1% over 5 years, respectively [14]. In contrast, these rates were lower than the 5-year cumulative incidence of ESKD of 5.2% in the United States [12]. A systematic review on a cohort of patients with LN demonstrated a cumulative incidence of ESKD at 5 years of 3–11%, 10 years of 6–19%, and 15 years of 19–25% [15]. These data showed that SLE patients are at high risk of ESKD, which causes poor quality of life and high mortality for these patients. Better management of SLE is needed to reduce the risk of ESKD in New Zealand.

Our study demonstrated that men are associated with an increased risk of ESKD in SLE patients. Men had twice the prevalence rate of ESKD than women and the HR of developing ESKD for men with SLE was 3.37 compared to women. There is discrepancy in other studies with some supporting this gender association with renal disease[16–19] and others not, though many of these demonstrated that men were susceptible but was not statistically significant [20, 21]. In Taiwan, the incidence of ESKD requiring renal replacement therapy in male SLE was 1157.0 (95% CI 502.4–1811.6) per 100,000 person-years compared to 545.8 (95% CI 388.1–703.5) per 100 000 person-years in female SLE patients [18]. A study with a Swedish multi-center SLE cohort demonstrated that men with SLE had an overall higher risk for progression into ESKD with an HR of 5.1 (95% CI 2.1–12.5) [17]. A large cohort study from the Spanish Society of Rheumatology Registry of Patients With Systemic Lupus Erythematosus (RELESSER) showed that the risk for LN development was 47.85% in male SLE patients compared to 30.91% in women [16].

Age at SLE diagnosis has been identified in previous studies as an important factor for increased risk of ESKD [15, 16, 19]. However, we could not find significant impact of age on risk of ESKD. This could be because of the limited number of new SLE patients and follow-up time in our study. However, we found that the highest prevalence of ESKD was found in the age group of 20–39 years and then decreased with age. This is consistent with other studies where SLE patients with ESKD/LN were younger than SLE patients without ESKD/LN [15, 16, 19]. Compared to patients of 50 years or older, the ORs for developing LN in patients aged less than 16 years old and patients aged 16–50 years were 6.06 (95% CI 4.29–8.56) and 2.52 (95% CI 1.91–3.32 [16].

Māori and Pacific people with SLE had higher incidence and prevalence of ESKD than others in our study. These ethnicities were also more likely to be younger with ESKD with 64.8% and 60.3% being less than 40 years. Burling et al.[8] in 2006 also found that Māori and Pacific patients with SLE had a higher risk of developing LN, with an odds ratio of 8.47 and 3.11 compared to others. Asian patients had a slightly higher prevalence of ESKD than European/others, but the difference in developing ESKD was not significant between these two groups. Ethnic difference in risk of ESKD/LN among SLE patients was also presented in overseas studies. Multiple studies have demonstrated that Hispanic, African American and Asian patients with SLE are more likely to get ESKD/LN with worse outcomes [15, 19–21]. Platinga et al. [21] demonstrated incidence rates of ESKD were 13.8 per 1000 patient-years for African Americans with SLE and 3.3 for white patients. In a cohort of 1827 SLE patients, LN was found 31.0% of patients at enrollment, and this rate was 49.3% for Hispanic, 39.9% in African, 36.8% in Asian, and 20.3% in Caucasian [19].

Māori patients with ESKD had twice the risk of all-cause mortality compared to European/others. This is also found in a New Zealand retrospective cohort study involving adults who commenced treatment for ESKD in 2002–2011 [22]. This study showed that New Zealand European patients experienced lower mortality than Māori patients (age-standardized mortality rate ratio 0.58, 95% CI 0.51–0.67) [22]. Older patients also had higher mortality than younger patients which means that the survival time from ESKD diagnosis was longer for younger patients than older patients. However, the age at death for the younger patients was still much lower than the older age groups. For example, the mean age at death was only 21 years old for patients diagnosed with ESKD under the age of 20 years old and was 42 years for patients diagnosed at the age of 20–39 years.

This is the first population-based study in SLE-related ESKD in New Zealand, using data linkage of the national administrative datasets with the ANZDATA registry data. The ANZDATA data are a comprehensive dataset that includes the detailed information on ESKD including primary disease. This study also has a few limitations. The ANZDATA only records ESKD cases having renal replacement treatment, and ESKD patients not treated were not recorded though the number should be small. Therefore, the number of ESKD patients was probably underestimated. The follow-up time for examining the risk of developing ESKD among new SLE patients was relatively short, which resulted in unsignificant HR for some important factors including age and Asian ethnicity.

## Conclusions

SLE patients had a high prevalence rate of ESKD. Men, Māori, and Pacific patients with SLE were more likely to develop ESKD. Māori patients with ESKD had poorer survival than other patients. Interventions are needed to reduce the risk of ESKD and to improve the survival of ESKD patients for the disadvantaged groups.

## Data Availability

The data used for this study are not publicly available because of the ethics for patient information. They can be access through the Ministry of Health and the Australia and New Zealand Dialysis and Transplant Registry with appropriate ethics approval.
